# Volunteering and instrumental support during the first phase of the pandemic in Europe: the significance of COVID-19 exposure and stringent country’s COVID-19 policy

**DOI:** 10.1186/s12889-023-17507-5

**Published:** 2024-01-05

**Authors:** Septi Kurnia Lestari, Malin Eriksson, Xavier de Luna, Gunnar Malmberg, Nawi Ng

**Affiliations:** 1 Centre for Demographic and Ageing Research, Umeå University, Umeå, Sweden; 2 Department of Epidemiology and Global Health, Umeå University, Umeå, Sweden; 3 Department of Social Work, Umeå University, Umeå, Sweden; 4 Umeå School of Business, Economics and Statistics, Umeå University, Umeå, Sweden; 5 Department of Geography, Umeå University, Umeå, Sweden; 6 School of Public Health and Community Medicine, Institute of Medicine, University of Gothenburg, Gothenburg, Sweden

**Keywords:** COVID-19, Social support, Social participation, Volunteering, Older population, SHARE, Europe, Ageing population

## Abstract

**Background:**

The COVID-19 control policies might negatively impact older adults’ participation in volunteer work, instrumental support provision, and the likelihood of receiving instrumental support. Studies that quantify changes in these activities and the related factors are limited. The current study aimed to examine the level of volunteering, instrumental support provision and receipt before and during the first phase of the COVID-19 pandemic in Europe and to determine whether older adults’ volunteering, instrumental support provision and receipt were associated with individual exposure to COVID-19 and the stringency of country’s COVID-19 control policy during the first phase of the COVID-19 pandemic.

**Methods:**

A cross-sectional survey using data from the Survey of Health, Ageing and Retirement in Europe (SHARE) Corona Survey 1 was designed to focus on community-dwelling Europeans aged ≥50 years. History of participation in volunteering work and instrumental support provision or receipt was assessed from the previous SHARE Wave data. The country’s COVID-19 control policy stringency index (S-Index) was from the Oxford COVID-19 Government Response Tracker database. A total of 45,669 respondents from 26 European countries were included in the volunteering analysis. Seventeen European countries were included in the analyses of instrumental support provision (N = 36,518) and receipt (N = 36,526). The multilevel logistic regression model was fitted separately to analyse each activity.

**Results:**

The level of volunteering and instrumental support provision was lower during the pandemic, but instrumental support receipt was higher. The country S-Index was positively associated with support provision (OR:1.13;95%CI:1.02–1.26) and negatively associated with support receipt (OR:0.69;95%CI:0.54–0.88). Exposure to COVID-19 was positively associated with support receipt (OR:1.64;95%CI:1.38–1.95). COVID-19 exposure on close ones positively associated with volunteering (OR:1.47;95%CI:1.32–1.65), support provision (OR:1.28;95%CI:1.19–1.39), and support receipt (OR:1.25;95%CI:1.15–1.35).

**Conclusions:**

The COVID-19 pandemic impacted older Europeans’ volunteering, instrumental support provision, and instrumental support receipt from outside their household. When someone close to them was exposed to COVID-19, older Europeans were likely to receive instrumental support and to volunteer and provide instrumental support. A stricter country’s COVID-19 control policy might motivate older adults to provide instrumental support, but it prevents them from receiving instrumental support from outside their households.

**Supplementary Information:**

The online version contains supplementary material available at 10.1186/s12889-023-17507-5.

## Background

The COVID-19 pandemic has had unprecedented impacts on various facets of people’s lives. Besides the virus’s direct health implications, the pandemic mitigation measures, such as physical distancing, stay-at-home orders, travel limitations, and area lockdowns, have negatively affected social interactions [1]. Moreover, COVID-19 restrictions and financial constraints compelled volunteer organisations to either suspend or scale down their operations [2, 3].

This study assesses changes in older adults’ participation in volunteer work and social support and how COVID-19 exposure (have COVID-19 symptoms, have tested positive, or have been hospitalised due to COVID-19) and the stringent country’s COVID-19 policy may be associated with European older adults’ receipt of instrumental support and participation in volunteering and instrumental support provision.

The COVID-19 pandemic has had a more pronounced impact on the ability of older adults to volunteer and provide or receive instrumental support than their younger counterparts. This is due to the heightened susceptibility of the older population to severe COVID-19 outcomes and fatalities, which is exacerbated by pre-existing health conditions and age-related physiological decline. Therefore, stricter adherence to COVID-19 control policies, notably the stay-at-home order, has been advised for this population [4, 5]. The imposition of COVID-19 restrictions has led to disruptions in the support networks and care exchanges of older adults with individuals outside their households [1], making those who rely on external assistance even more vulnerable [6]. The pandemic has also made it challenging for older adults who volunteered before the pandemic to continue their contributions. Moreover, the disturbance in social interactions and the exchange of social support could detrimentally impact the mental health [7] and overall quality of life [8] of older adults.

Studies have shown that a substantial share of the older European population provided social support and participated in volunteer work before the pandemic. A study showed that about a fifth of Europeans aged 50 and over received support from people outside the household [9]. A third of older European adults provided instrumental support (such as personal care, help related to paperwork, or household chores) for people outside their households [10]. An even larger share of older adults helped care for their grandchildren [11, 12]. Older adults were also active in volunteer work, e.g., 34% in the Netherlands, 29% in Denmark, 28% in Switzerland, 8% in Estonia, 7% in Czechia, and 5% in Spain [13].

During the early phase of the pandemic (spring-summer 2020), about 20% of older adults in 26 European countries had difficulties obtaining support from outside their households [14]. Nonetheless, studies from various countries, including Canada, the USA [15], the UK [16] and Sweden [17] reported older adults’ involvement in volunteering and support provision despite the COVID-19 restrictions. However, the levels of instrumental support receipt, instrumental support provision, and volunteering during the first phase of the pandemic remain unclear.

Also, further investigation on the determinants of volunteering, instrumental support provision and receipt during the pandemic is needed. Among the various potential determinants, individual COVID-19 exposure and the country’s COVID-19 policy are less explored [18]. Understanding the impacts of COVID-19 exposure and the country’s COVID-19 policy on older adults’ instrumental support receipt and engagement in volunteer work and instrumental support provision can aid in designing more effective mitigation strategies for future pandemics, ensuring minimal negative impacts on older adults’ social support networks.

Building upon prior studies, the current study aims to examine the level of volunteering, instrumental support provision and receipt before and during the first phase of the COVID-19 pandemic and to determine whether older adults’ volunteering, instrumental support receipt and provision were associated with individual exposure to COVID-19 and the stringency of country’s COVID-19 control policy during the first phase of the COVID-19 pandemic in Europe.

## Methods

### Data sources

#### The survey of health, ageing and retirement in Europe (SHARE)

The primary data sources of this study were the SHARE Corona Survey 1 (SCS1) [19], SHARE Wave 7 [20], and SHARE Wave 6 [21]. SHARE is a cross-national panel database of microdata on socioeconomic, social, and family networks and the health of individuals aged 50 and over. SHARE has collected data approximately every two years since 2004 [22]. In 2020, the COVID-19 outbreak in Europe halted the regular SHARE Wave 8 data collection. In response to the pandemic, the SCS1 was conducted between June and September 2020. It collected data on the changes in the socioeconomic situation, health and health behaviour, mental health, changes in social networks, COVID-19-related symptoms, and healthcare service use during the first phase of the pandemic. Unlike the regular SHARE, interviews in the SCS1 were conducted via telephone instead of face-to-face [23]. Detailed information regarding the SHARE survey method is available elsewhere [22, 23].

#### Coronavirus government response tracker

The Oxford COVID-19 Government Response Tracker (OxCGRT) is a publicly accessible dataset containing data on COVID-19 policy measures from over 180 countries. The record starts on 1 January 2020 and is continuously updated. Detailed data collection and processing methods have been published elsewhere [24].

#### The centre for systems science and engineering

The countries’ total confirmed COVID-19 cases per million used in the present study were obtained from the Centre for Systems Science and Engineering at Johns Hopkins University [25]. This data is available on the Our World in Data website (ourworldindata.org).

### Outcome variables

The primary outcome variables in this study were volunteering, instrumental support provision, and instrumental support receipt during the first phase of the pandemic. Participation in volunteer work was determined from the question, “Since the outbreak of Corona, did you do any other volunteering activity?”. Instrumental support receipt was determined from the question, “Since the outbreak of Corona, were you helped by others from outside of the home to obtain necessities, e.g., food, medications or emergency household repairs?”. Similar questions were used to determine instrumental support provision.

### Explanatory variables

*The country’s COVID-19 control policy stringency index* (S-Index) was calculated from eight containment and closure policies (i.e., schools, workplaces, and public transport closures, public events cancellation, limitations on gatherings, restrictions on local and international travel, and orders to “shelter-in-place”) and one health policy indicator (i.e., record presence of public info campaigns). This index ranged from 0 to 100, with a higher index indicating stricter containment policies [24]. In this study, the country’s stringency index was the average of individual-level stringency indexes in each country.

The individual-level average stringency index was the sum of daily stringency indexes from 11 March 2020 (the date of WHO’s declaration of the COVID-19 pandemic) until the end of the interview month of each respondent, divided by the number of days elapsed between the two dates. The two-time points were chosen because most questions in SCS1 asked about conditions “since the COVID-19 outbreak” and because SHARE only recorded the month and year of the interview.

*The COVID-19 exposure status of respondents and their close ones* (i.e., family, friends, or neighbours) were determined by asking the respondents whether they or their close ones have ever experienced COVID-19 symptoms, have tested positive, or have been hospitalised due to COVID-19.

### Control variables

The country’s total COVID-19 cases per million was the average of the total COVID-19 cases per million on each respondent’s last date of the interview month. For example, the total COVID-19 cases per million on 30 June 2020 were assigned to respondents interviewed in June 2020. The country’s volunteering level before the pandemic was calculated based on SHARE Wave 7 data. The country’s levels of providing and receiving instrumental support before the pandemic were calculated based on SHARE Wave 6 data. The individual-level control variables are described in Table 1.

### Statistical analyses and analytical sample

Inclusion criteria for the study sample were respondents aged 50 and over, who never resided in a nursing home, and who had complete data on variables required for the analysis. The current study analysed each outcome separately. A total of 51,264 respondents had data on at least one of the three outcome variables before and during the first phase of the pandemic. However, they had missing data in some explanatory and control variables. Thus, different sub-samples were constructed to retain the maximum number of samples in the analyses (see Supplementary Figure A1).

Weighted descriptive analyses were performed to assess the individual characteristics and the level of volunteering, providing instrumental support, and receiving instrumental support by the individual characteristic. A maximum of 51,264 respondents were included in this analysis. Next, the levels of volunteering (N = 47,332), instrumental support provision (N = 37,820), and instrumental support receipt (N = 37,828) before and during the first phase of the pandemic were analysed.

The multilevel logistic regression analyses were separately conducted with volunteering (N = 45,669), instrumental support provision (N = 36,518), and instrumental support receipt (N = 36,526) as the outcomes. Random intercept models were fitted with the country as the grouping variable. Thus, intercept may vary across countries, while the effects of explanatory variables were assumed to be the same for all countries. All models follow the general equation as presented in Eq. 1. Where *β*_*00*_ (overall intercept) is the log-odds that the outcome is equal to one when the other parameters are equal to zero. *u*_*0j*_ is the country’s random effect. *xij* is the value of individual-level explanatory variable x for individual *i* in country *j*, while *vj* is the value of country-level explanatory variable *v* for individuals in country *j. β*_*1*_ and *β*_*01*_ are the effect of one unit change of variable x and v, respectively, on the log-odds that the outcome is equal to one when *u* is held constant [26].

**Table 1:** Operational definition of the control variables

Variables	Definitions and categories
*Sociodemographic*	
Sex	*Man* and *woman.*
Age group	*50–59*, *60–69*, *70–79*, and ≥ *80.*
Education level	*Low* (ISCED 0/1/2), *middle* (ISCED 3 – upper secondary education/ISCED 4 – post-secondary non-tertiary), and *high* (ISCED 5/6).
Changes in employment status	*Unemployed* (unemployed at the time of COVID-19 outbreak), *became* unemployed (employed/self-employed/family business at the time of but was unemployed after the COVID-19 outbreak), *stayed employed* (employed at the time of- and after COVID-19 outbreak).
Good household economic status	Since the COVID-19 outbreak, the household has made ends meet *fairly easily* or *easily*.
*Social relations during the pandemic*	
Lived alone	Household size equal to one.
Frequent social contacts	Reported *daily, several times a week, or about once a week* direct and/or indirect (by phone, email, or any other electronic means) contact with people outside the home.
*Health-related factors before the COVID-19 outbreak*	
Self-rated health	*Good* (excellent/very good/good health) or *poor* (fair/poor health).
Had a history of chronic condition(s)	Reported at least one of the following: hip fracture, diabetes, heart attack, chronic lung disease, or cancer, based on SHARE Wave 7 data.
Received formal care	Reported that they received home.
*Health-related factors after the COVID-19 outbreak*	
Changes in self-rated health	*Improved*, *worsened*, or *about the same*.
Had new chronic condition(s)	Reported at least one of the following: hip fracture, diabetes, heart attack, chronic lung disease, or cancer, based on SCS1 data.
Feeling anxious	Reported feeling nervous, anxious, or on edge in the last month.
Feeling sad or depressed	Reported feeling sad or depressed in the last month.
*History of the outcomes before the pandemic*	
Volunteering	In the last 12 months, done voluntary or charity work (Wave 7).
Instrumental support provision	In the last 12 months, provided help (personal care, help related to paperwork, or household chores) for any family member from outside the household, friend, or neighbour (Wave 6).
Instrumental support receipt	In the last 12 months, received help (personal care, help related to paperwork, or household chores) from any family member from outside the household, friend, or neighbour (Wave 6).

Eq. 1 $${\rm{log}}\left( {\frac{{{{\rm{\pi }}_{{\rm{ij}}}}}}{{{\rm{1 - }}{{\rm{\pi }}_{{\rm{ij}}}}}}} \right) = {\beta _{00}} + {\beta _1}{x_{ij}} + {\beta _{01}}{v_j} + {u_{0j}}$$

We specified four multilevel logistic regression models for each outcome. The first was the null model, which included the outcome variable only. All individual- and country-level control variables were added in the second model. The COVID-19 exposure variables were added in the third model, and the standardised country’s S-Index in the final (fourth) model.

## Results

### Study sample characteristics

Slightly more than half of the study sample were women. About 36% were aged 60–69 years, and about 42% had middle education levels. Around 25% were employed before and during the pandemic, and about 7% became unemployed during the pandemic. During the first phase of the pandemic, 67% had a good household economic status, 26% lived alone, and 65% had frequent direct or online contact with people outside the home.

Regarding health status, 66% reported good self-rated health before and during the pandemic. Only a small share of the study sample had worsened self-rated health. Around 36% reported at least one chronic condition before the pandemic, and about 5% reported new chronic conditions during the pandemic. About 30% of study samples reported feeling anxious, and a similar share of respondents reported feeling depressed during the pandemic. Around 3% were exposed to COVID-19, and 16% reported that their close ones were exposed to COVID-19 (see Table 2).

**Table 2:** Individual characteristics and levels of volunteering, providing instrumental support, and receiving instrumental support during the first phase of the pandemic by individual’s characteristics (% and its 95% CI)

	Respondent characteristics	Level of volunteering		Level of providing instrumental support		Level of receiving instrumental support		
Sex								
Man	45.8 (44.7–46.9)	5.5 (4.8–6.2)		20.2 (18.7–21.8)		16.5 (15.2–17.8)		
Woman	54.2 (53.1–55.3)	5.3 (4.7-6.0)		21.3 (20.2–22.5)		27.9 (26.9–28.9)		
Age group								
50–59	25.4 (24.3–26.5)	5.3 (4.3–6.5)		32.9 (30.3–35.6)		7.6 (6.2–9.2)		
60–69	36.4 (35.4–37.5)	6.7 (5.8–7.6)		24.4 (22.8–26.0)		13.8 (12.4–15.4)		
70–79	22.4 (21.7–23.0)	5.5 (4.9–6.2)		12.3 (11.5–13.3)		31.6 (30.4–32.8)		
80+	15.9 (15.3–16.4)	2.5 (2.0-3.2)		5.3 (4.5–6.1)		54.6 (52.9–56.4)		
Educational level								
Low	35.6 (34.5–36.6)	2.7 (2.1–3.4)		12.9 (11.4–14.5)		29.0 (27.4–30.7)		
Middle	42.3 (41.3–43.4)	5.3 (4.6–6.1)		23.8 (22.4–25.2)		20.5 (19.4–21.6)		
High	22.1 (21.2–23.0)	10.0 (8.9–11.3)		27.9 (25.7–30.1)		16.5 (15.2–17.9)		
Conditions before the pandemic								
Had chronic condition(s)								
Yes	35.9 (35-36.9)	4.7 (4.1–5.5)		15.4 (14.2–16.6)		32.3 (31.0-33.5)		
No	64.1 (63.1–65)	5.7 (5.2–6.4)		23.9 (22.6–25.2)		17.3 (16.3–18.4)		
Received home care								
Yes	5.1 (4.8–5.5)	1.7 (1.1–2.6)		8.0 (6.4–10.0)		66.6 (63.3–69.7)		
No	94.9 (94.5–95.2)	5.6 (5.1–6.1)		21.5 (20.5–22.5)		20.3 (19.5–21.1)		
Conditions during the pandemic								
Had new chronic condition(s)								
Yes	5.0 (4.7–5.4)	4.5 (3.1–6.4)		12.6 (10.4–15.3)		37.3 (34.0-40.6)		
No	95.0 (94.6–95.3)	5.4 (5.0-5.9)		21.2 (20.3–22.2)		21.9 (21.1–22.7)		
Frequent social contacts								
Yes	65.1 (64.0-66.1)	6.0 (5.4–6.6)		25.0 (23.8–26.3)		23.9 (22.9–24.8)		
No	34.9 (33.9–36.0)	4.3 (3.7-5.0)		13.0 (11.8–14.3)		20.5 (19.0–22.0)		
Feeling anxious								
Yes	30.6 (29.6–31.6)	4.6 (4.0-5.4)		21.3 (19.5–23.2)		29.0 (27.2–30.8)		
No	69.4 (68.4–70.4)	5.7 (5.2–6.4)		20.6 (19.6–21.7)		19.8 (19.0-20.7)		
Feeling sad or depressed								
Yes	28.9 (27.9–29.8)	5.2 (4.3–6.2)		19.8 (18.1–21.7)		32.5 (30.9–34.2)		
No	71.2 (70.2–72.1)	5.5 (5.0–6.0)		21.2 (20.1–22.3)		18.6 (17.7–19.6)		
Good household economic status								
Yes	67.4 (66.4–68.4)	6.6 (6.0-7.3)		22.7 (21.5–23.9)		21.5 (20.6–22.4)		
No	32.6 (31.6–33.6)	3.0 (2.5–3.7)		17.7 (16.2–19.3)		25.0 (23.3–26.8)		
Lived alone								
Yes	26.5 (25.6–27.4)	6.3 (5.3–7.5)		19.5 (17.8–21.3)		38.6 (36.9–40.4)		
No	73.5 (72.6–74.4)	5.1 (4.6–5.6)		21.3 (20.2–22.4)		16.9 (16.0-17.8)		
Changes after the outbreak								
Employment status								
Not employed	67.6 (66.5–68.7)	4.7 (4.3–5.2)		14.9 (14.1–15.7)		30.1 (29.1–31.1)		
Became unemployed	6.8 (6.2–7.5)	7.0 (5.0-9.8)		35.5 (30.8–40.4)		9.9 (7.2–13.4)		
Stayed employed	25.6 (24.5–26.7)	6.7 (5.6–7.9)		32.5 (30-35.2)		6.5 (5.3-8.0)		
Self-rated health								
Poor-improved	1.8 (1.5–2.1)	6.0 (3.2–11.0)		18.5 (13-25.8)		33.0 (26.6–40.1)		
Poor-worsened	5.1 (4.7–5.5)	2.1 (1.4–3.2)		9.9 (7.7–12.6)		44.6 (41.0-48.2)		
Poor-same	21.6 (20.8–22.5)	2.5 (2.0-3.1)		11.2 (9.9–12.7)		37.1 (34.9–39.3)		
Good-improved	1.3 (1.1–1.5)	9.7 (6.4–14.4)		31.0 (24.5–38.4)		13.3 (10.0-17.5)		
Good-worsened	4.0 (3.7–4.4)	10.1 (7.0-14.4)		21.6 (17.9–25.9)		30.4 (26.6–34.6)		
Good-same	66.2 (65.2–67.2)	6.2 (5.6–6.9)		24.6 (23.4–25.9)		15.7 (14.9–16.5)		
COVID-19 exposure								
on close ones								
Yes	16.3 (15.4–17.3)	9.0 (7.6–10.6)		29.2 (26.4–32.1)		21.4 (18.6–24.4)		
No	83.7 (82.7–84.6)	4.7 (4.2–5.2)		19.2 (18.2–20.2)		22.9 (22.1–23.7)		
on respondent								
Yes	3.2 (2.7–3.7)	9.0 (5.9–13.6)		21.2 (16.7–26.7)		22.7 (17.9–28.4)		
No	96.8 (96.3–97.3)	5.3 (4.8–5.8)		20.8 (19.9–21.8)		22.7 (21.9–23.5)		

Note: weighted data

Supplementary Table A1 presents country characteristics, including the S-Index, total COVID-19 cases, levels of volunteering, and instrumental support provision and receipt before the pandemic. Denmark had the highest (36.7%) level of volunteering, while Bulgaria had the lowest (3.5%). Instrumental support provision was lowest in Spain (9.0%) and highest in Denmark (57.8%). Czechia (37.2%) had the highest level of instrumental support receipt, in contrast with Portugal (10.1%). In our analytical sample, France had the highest country’s S-Index (around 75). The lowest was around 51 in Finland (analytical sample of volunteering) or around 55 in Luxembourg (analytical sample of instrumental support). The country’s COVID-19 cases ranged from around 356 per million in Slovakia (analytical sample of volunteering) to around 431 per million in Greece (analytical sample of instrumental support) to about 10,000 per million in Luxembourg.

### Levels of volunteering, providing instrumental support, and receiving instrumental support before and during the pandemic

**Fig. 1:**
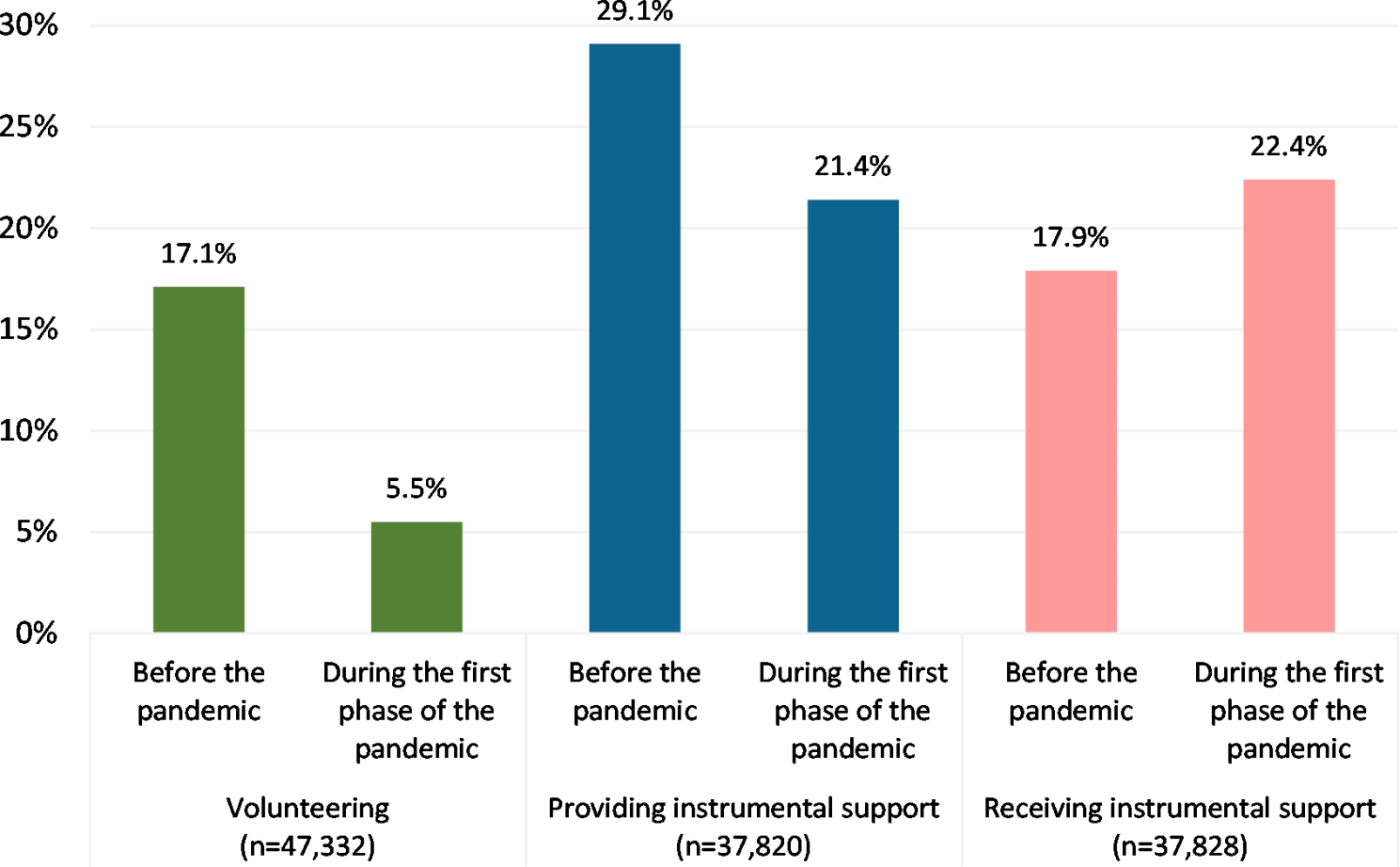
Levels of volunteering, providing instrumental support, and receiving instrumental support before and during the first phase of the pandemic. Note: Weighted data

Before the pandemic, 17.1% of respondents participated in volunteer work, but only 5.5% did so during the first phase of the pandemic. About 29.1% of the study sample provided instrumental support before the pandemic, which declined to 21.4%. On the contrary, 17.9% of study samples received instrumental support before, which increased to 22.4% during the pandemic (See Fig. 1).

During the first phase of the pandemic, higher levels of volunteering and providing instrumental support were observed among healthy people, those with higher education levels, good household economic conditions, and those employed before the pandemic, regardless of their employment status after the COVID-19 outbreak. Those in younger age groups had higher levels of providing instrumental support. On the contrary, a higher level of receiving instrumental support was found among the older age groups, those with lower education levels, those not employed, those who lived alone, and those who had poorer health (see Table 2).

### COVID-19 exposure and stringent country’s COVID-19 control policy as determinants of volunteering, instrumental support provision, and instrumental support receipt

The ICC (Intraclass Correlation Coefficient) from the null model indicated that the between-country differences explained 19.4% variations in the chances of volunteering, 5.1% variations in the chances of instrumental support provision, and 6.0% of variations in the chances of instrumental support receipt. These findings justify the use of multilevel logistic regression analysis in this study. The ICC became smaller when the explanatory variables were added. The ICC in the final model of each outcome is 5.9% for the volunteering model, 1.2% for providing instrumental support, and 5.0% for receiving instrumental support (Supplementary Table A2-A4).

**Fig. 2:**
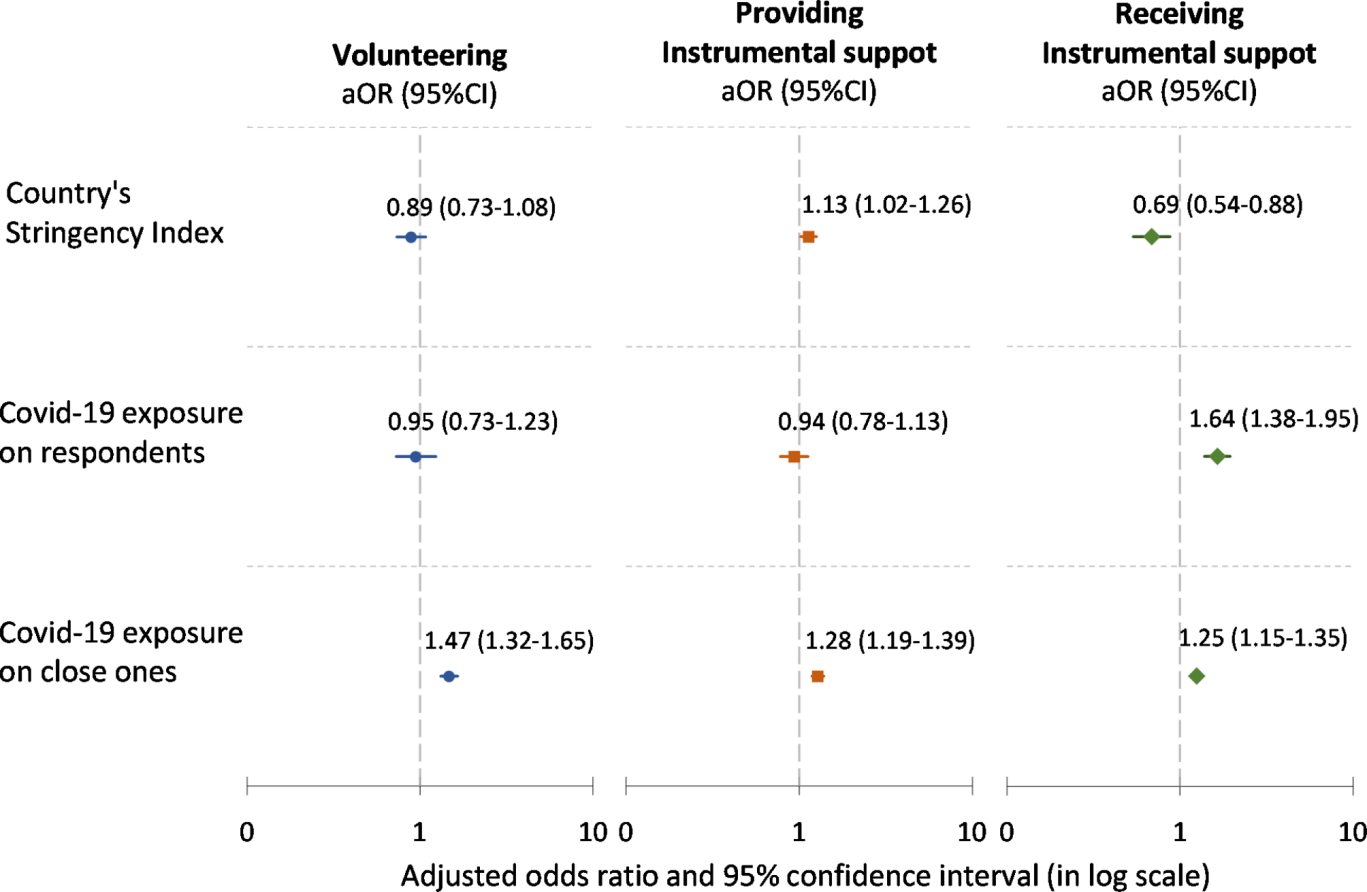
Results from multilevel logistic regression analysis of volunteering (N = 45,669), providing support (N = 36,518), and receiving support (N = 36,526) during the first phase of the pandemic (adjusted odds ratio and its 95%CI). Notes: Each model was adjusted for sex, age, education level, employment status, household economic status, living alone, frequent contact, history of chronic conditions (heart attack, cancer, hip fracture, diabetes, and chronic lung disease), presence of new chronic conditions, changes in self-reported health, self-rated depression, self-reported anxiety, history of receiving home care before the pandemic, country’s level of volunteering, providing instrumental support, or receiving instrumental support before the pandemic, and the country’s total of COVID-19 cases per million

The final multilevel models show that a 7-unit (one standard deviation) increase in the country’s S-Index was associated with 13% higher (OR:1.13, 95%CI:1.02–1.26) odds of support provision and 31% lower (OR:0.69, 95%CI:0.54–0.88) odds of support receipt. However, the country’s S-Index was not associated with the odds of volunteering (OR:0.89, 95%CI:0.73–1.08). Furthermore, older adults exposed to COVID-19 were more likely (OR:1.64, 95%CI:1.38–1.95) to receive support during the pandemic. This exposure status was not associated with the odds of volunteering and providing support. However, older adults whose close ones were exposed to COVID-19 were more likely to engage in volunteer work (OR:1.47, 95%CI:1.32–1.65), provide instrumental support (OR:1.28, 95%CI:1.19–1.39), and receive instrumental support (OR:1.25, 95%CI:1.15–1.35) (See Fig. 2).

## Discussion

As expected, the overall levels of providing support and volunteering were lower during the first phase of the pandemic than before the pandemic. However, the level of receiving support was slightly higher during the first phase of the pandemic. The present study further focused on examining whether individual exposure to COVID-19 and the stringency of the country’s COVID-19 control policy during the first phase of the COVID-19 pandemic in Europe were associated with older adults’ volunteering, instrumental support provision and receipt using multilevel logistic regression analyses.

Individual COVID-19 exposure was found to be associated with older European adults’ receipt of instrumental support and participation in volunteer work and instrumental support provision. When older adults or their close ones were exposed to COVID-19, their need for support was likely to increase. We found that older Europeans were more likely to receive support from outside the household when they or their close ones were exposed to COVID-19. These findings suggest that older European’s social support networks reacted to their increased needs due to COVID-19, which aligned with previous studies [15, 27]. On the other hand, we found that older adults also reacted to other people’s increased need for support. Older Europeans were more likely to volunteer or provide instrumental support when their close ones were exposed to COVID-19. Older adults’ engagement in support provision and volunteering is primarily driven by their altruistic values and belief in familial and social obligation [28, 29]. Therefore, when they perceive their close ones or other people in their community struggling due to the COVID-19 crisis, they provide help directly or by joining volunteer work.

On the country level, the stringency of the country’s COVID-19 control policy (e.g., area lockdown and travel restrictions) might negatively affect social interaction, including volunteering and instrumental support exchange with people outside the household [30]. Unsurprisingly, we found a negative association between the stringency of the country’s COVID-19 control policy and receiving instrumental support. Our finding suggests that when the COVID-19 restriction was more intense, the regular support providers who lived far away had fewer opportunities to support older adults. Hence, older adults were less likely to receive instrumental support.

However, we also found that older adults were more likely to provide instrumental support for people outside their households when the country’s COVID-19 control policy was stricter. In this case, the strict COVID-19 policy may indicate other people’s increased need for support, prompting support provision by older adults. Older Europeans’ primary beneficiaries of instrumental support (outside the household) were their parents or children [10], who were likely to live close by [31]. Hence, lockdown restrictions may not negatively affect support provision because the support recipients live nearby. In addition, a study in Europe observed that mobility related to non-necessary (recreation, transport, and work) activities generally decreases with the increasing S-Index. Mobility related to necessary activity, such as going to the grocery or drugstore, was also decreasing but not as steep as non-necessary activity [32]. Thus, the COVID-19 restrictions might not prevent older adults from providing instrumental support outside the home if they consider it necessary.

As for volunteering, unexpectedly, in the present study, the stringency of the country’s COVID-19 control policy and individual COVID-19 exposure was not associated with the likelihood of volunteering. The possible explanation is that many volunteer organisations adapted to the pandemic control policies by transforming or complementing their volunteer activity with online activities [33]. As people could engage in virtual volunteering from their homes [34], the lockdown policy and their COVID-19 exposure status may have little to no effect on this activity. Unfortunately, in the SHARE questionnaire, there was no indication of the type of voluntary activity. Thus, we could not ascertain whether our findings resulted from a mixed association with different volunteering types.

### Strengths and limitations

The current studies have several strengths. The SHARE data we used were collected per the standard design, interview method, instrument, and quality assurance procedure to ensure comparability across countries. Furthermore, we employed several analytical strategies. Multilevel logistic regression analyses used in the present study separated the contextual effect, i.e., country, from the individual-level effect. The country’s level and the individual’s history of volunteering, providing instrumental support, and receiving instrumental support were controlled for. These measures resulted in more valid estimates of the association between the variable of interest (individual exposure to COVID-19 and the stringency of the country’s COVID-19 control policy) and older adults’ volunteering, instrumental support provision and receipt during the first phase of the COVID-19 pandemic.

Despite the strengths, we acknowledged that this study also had limitations. Variables used in the present study were limited by their availability in the SHARE datasets. It may be disadvantageous to use a history of volunteering from Wave 7 and support receipt and provision from Wave 6. The SHARE Corona Survey 1 was conducted five years following Wave 6 or three years following Wave 7. During this wide time gap, older adults may stop providing support or start receiving support [10, 35]. Also, many respondents aged 50–54 in 2020 did not have data before the pandemic as they were not eligible to participate in the previous waves. Thus, they had to be excluded from the analyses. As a result, our findings may not be representative of this age group.

COVID-19 exposure, support exchange, and volunteering data were collected simultaneously (in the SCS1). Thus, it is possible that some individuals reported instrumental support provisions that occurred before, after, or during their exposure to COVID-19. In the same vein, in calculating the country S-Index and total COVID-19 cases for the present analysis, we assumed that the reported engagement in volunteering and instrumental support receipt or provision occurred around the interview date. This assumption may not hold as the SHARE questions used a recall period of “since the COVID-19 outbreak”. Also, the SHARE data used in this study were self-reported, which might be under or overreported.

While the results of this study should be interpreted with caution, they nevertheless add valuable information to the body of knowledge on solidarity and active ageing in Europe during the first phase of the pandemic. Future studies can build upon and extend this work in several ways. A well-designed longitudinal study with appropriate instruments is required to investigate the causal effect of COVID-19 restrictions on volunteering and support exchange. Future studies should include indicators of national wealth, social inequality, policies regarding social protection and volunteering in each country, and how those policies have changed due to the pandemic. A comprehensive analysis of those contextual factors is also necessary to determine their impact on the different types of support provision and volunteering during the pandemic.

## Conclusion

The present study demonstrates that the COVID-19 pandemic affected European older adults’ instrumental support receipt and participation in volunteer work and instrumental support provision. During the first phase of the pandemic, European older adults showed solidarity by participating in volunteer work and providing instrumental support in response to others’ increased need for support due to COVID-19. They were also likely to receive instrumental support when they needed support due to COVID-19. The stringent country’s COVID-19 control policies might prevent older adults from receiving instrumental support from outside their households. Interestingly, older adults were likely to provide instrumental support for people living nearby during the stricter COVID-19 control policies. These findings show that a significant share of older European adults could provide informal help for others, even during a crisis. Thus, volunteer organisations, with support from the government, should tailor volunteer programs for older adults. Therefore, they can give their optimum contribution to distribute help, especially during a crisis such as the COVID-19 pandemic.

### Electronic supplementary material

Below is the link to the electronic supplementary material.

Supplementary Material 1: Study samples selection; Descriptive estimates of country-level variables by country; Complete results of multilevel logistic regression analyses

## Data Availability

SHARE data is distributed by SHARE-ERIC (Survey of Health, Ageing and Retirement in Europe – European Research Infrastructure Consortium) and is freely available to the scientific community after registration. All data users are subject to European Union and national data protection laws and the SHARE Conditions of Use. More details on data access are available at http://www.share-project.org/data-access.html.

## Abbreviations

COVID-19: Coronavirus Disease 2019
ICC: Intraclass Correlation Coefficient
SCS: SHARE Corona Survey
SHARE: Survey of Health, Ageing and Retirement in Europe
S-Index: Stringency Index
