# Supplementary material for: Volunteering and instrumental support during the first phase of the pandemic in Europe: the significance of COVID-19 exposure and stringent country’s COVID-19 policy
Source: BMC Public Health. 2024 Jan 5;24:99. doi: 10.1186/s12889-023-17507-5 (PMC10768320; doi:10.1186/s12889-023-17507-5)
Supplement: Supplementary file 1 — Supplementary Material 1: Study samples selection; Descriptive estimates of country-level variables by country; Complete results of multilevel logistic regression analyses [file 12889_2023_17507_MOESM1_ESM.pdf]

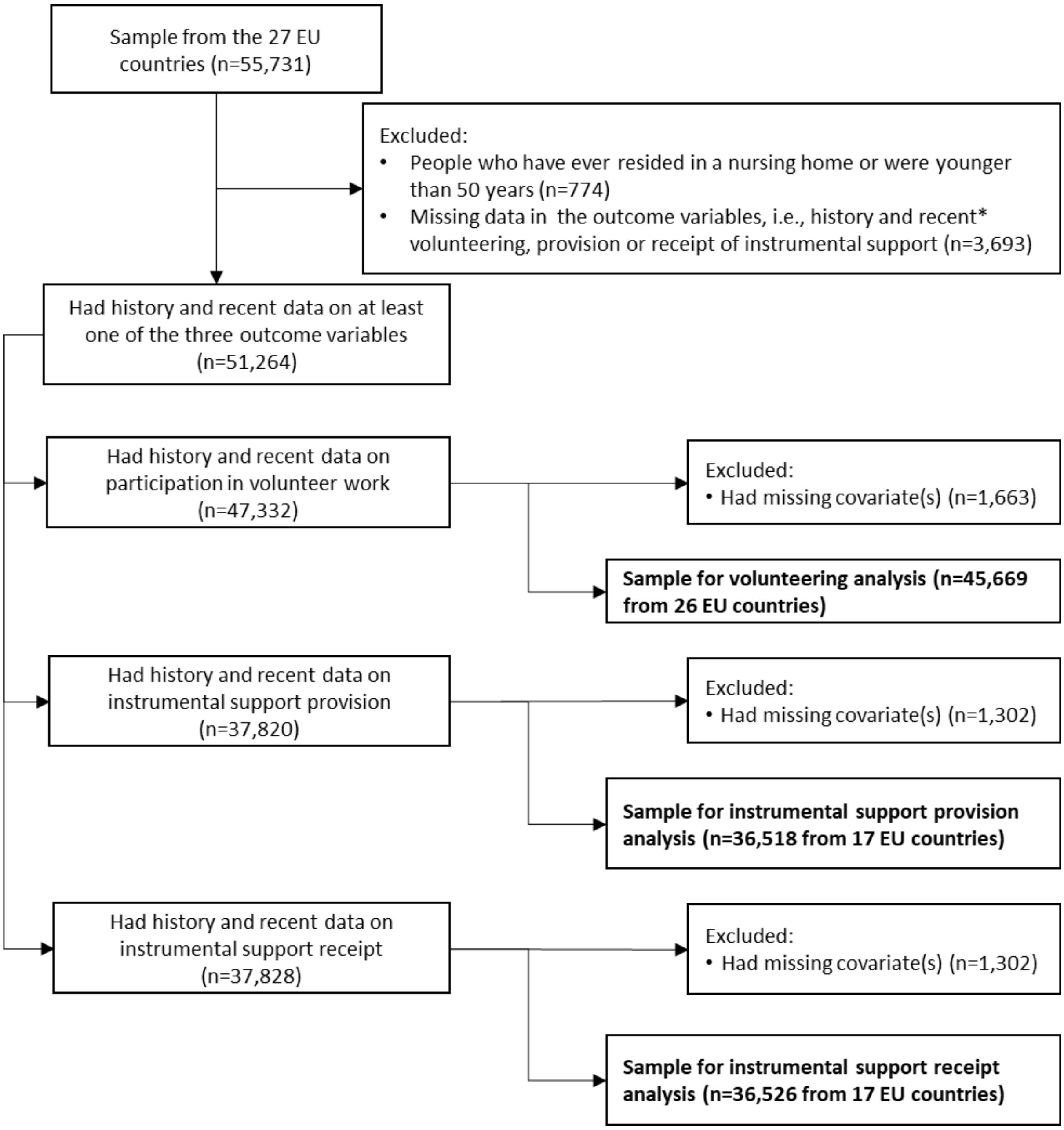

Note: SCS1 is SHARE Corona Survey 1

Figure A1. Study samples selection

Table A1. The country's COVID-19 control policy stringency index (S-Index), mean total COVID-19 per million people, and the levels of volunteering, providing instrumental support, and receiving instrumental support before the pandemic by country

Country	Sample for Volunteering analysis		Sample for providing instrumental support analysis		Sample for receiving instrumental support analysis		Volunteering (%)	Providing instrumental support (%)	Receiving instrumental support (%)
	S-Index	Mean total COVID-19 cases per million	S-Index	Mean total COVID-19 cases per million	S-Index	Mean total COVID-19 cases per million			
Austria	55.1	3,371.1	55.1	3,390.9	55.1	3,390.9	23.2	33.2	26.8
Belgium	69.2	5,533.9	69.1	5,537.4	69.1	5,537.4	30.0	42.1	18.2
Bulgaria	54.0	1,721.7					3.5		
Croatia	69.4	1,009.4	69.4	1,015.7	69.4	1,015.9	6.7	34.2	28.5
Cyprus	73.1	1,258.6					14.6		
Czechia	55.8	1,366.5	55.8	1,367.4	55.8	1,367.5	9.2	39.9	37.2
Denmark	64.6	2,308.8	64.6	2,309.7	64.6	2,309.8	36.7	57.8	29.0
Estonia	55.2	1,518.3	55.1	1,519.4	55.1	1,519.4	11.2	24.3	26.2
Finland	**51.1**	1,335.8					27.6		
France	**75.3**	3,155.2	**75.3**	3,156.2	**75.3**	3,156.2	27.5	39.5	16.7
Germany	64.9	2,414.5	64.9	2,415.0	64.9	2,415.0	27.8	39.4	23.6
Greece	65.4	433.3	65.4	**430.9**	65.4	**430.9**	8.4	19.0	17.9
Hungary	65.9	448.9					9.0		
Italy	73.9	4,029.9	73.9	4,031.5	73.9	4,031.5	11.9	19.2	12.0
Latvia	59.1	648.1					7.7		
Lithuania	63.8	692.3					5.7		
Luxembourg	55.0	**10,035.1**	**54.9**	**10,042.3**	**54.9**	**10,044.2**	28.1	24.7	11.4
Malta	67.4	1,844.8					17.8		
Poland	66.1	1,071.2	66.6	1,041.6	66.6	1,041.6	6.7	16.8	12.1
Portugal	73.9	4,700.2	73.8	4,747.4	73.8	4,747.4	9.9	17.7	10.1
Romania	69.7	1,662.7					3.9		
Slovakia	63.1	**356.4**					8.2		
Slovenia	60.5	883.5	60.4	886.3	60.4	886.3	13.0	23.0	15.1
Spain	71.3	5,601.8	71.2	5,625.3	71.2	5,625.1	5.3	9.0	10.6
Sweden	58.7	7,204.6	58.7	7,214.7	58.7	7,214.7	16.4	41.4	19.5
Switzerland	58.6	3,793.9	58.6	3,794.9	58.6	3,795.0	32.4	36.1	17.3

Table A2. Adjusted odds ratio (95% Confidence Interval) from multilevel logistic regression analysis of volunteering (N= 45,669)

	Model 1: null model	Model 2: + control variables	Model 3: + COVID-19 exposure	Model 4: Country S-Index
Intercept	0.03***(0.02-0.05)	0.01***(0-0.01)	0.01***(0-0.01)	0.01***(0-0.01)
Covid-19 exposure on close ones			1.47***(1.31-1.65)	1.47***(1.32-1.65)
Covid-19 exposure on respondents			0.95(0.73-1.23)	0.95(0.73-1.23)
Country's standardised S-Index				0.89(0.73-1.08)
Individual-level covariates				
Woman		0.87**(0.79-0.96)	0.87**(0.79-0.96)	0.87**(0.79-0.96)
Age group				
50-59				
60-69		1.11(0.95-1.31)	1.12(0.95-1.32)	1.12(0.95-1.32)
70-79		0.98(0.81-1.18)	0.99(0.82-1.19)	0.99(0.82-1.19)
80+		0.51***(0.4-0.65)	0.53***(0.42-0.67)	0.53***(0.42-0.67)
Educational level				
Low				
Middle		1.16*(1.02-1.33)	1.15*(1.01-1.32)	1.15*(1.01-1.31)
High		1.61***(1.4-1.84)	1.57***(1.37-1.8)	1.57***(1.37-1.8)
Good household SES		1.03(0.9-1.19)	1.02(0.89-1.17)	1.02(0.89-1.17)
Changes in employment status				
Unemployed				
Became unemployed		1.08(0.87-1.35)	1.06(0.85-1.32)	1.06(0.85-1.32)
Employed		1.04(0.91-1.19)	1.02(0.89-1.17)	1.02(0.89-1.16)
Had volunteering history		5.06***(4.6-5.58)	5.01***(4.55-5.52)	5.01***(4.55-5.52)
Living alone		1.07(0.96-1.2)	1.08(0.97-1.21)	1.08(0.97-1.21)
Had frequent direct/online contacts		1.28***(1.16-1.41)	1.28***(1.16-1.42)	1.28***(1.16-1.42)
Had chronic condition before x during the pandemic				
No x No				
No x Yes		1.07(0.78-1.46)	1.08(0.79-1.48)	1.08(0.79-1.48)
Yes x No		0.89*(0.8-0.99)	0.89*(0.8-0.99)	0.89*(0.8-0.99)
Yes x Yes		0.77(0.57-1.02)	0.77(0.57-1.02)	0.77(0.57-1.02)
Self-rated health before x during the pandemic				
Poor x Improved		1.56*(1.09-2.24)	1.55*(1.09-2.23)	1.55*(1.09-2.23)
Poor x Worsened		1.12(0.82-1.53)	1.11(0.81-1.52)	1.11(0.81-1.53)
Poor x Same				

	Model 1: null model	Model 2: + control variables	Model 3: + COVID-19 exposure	Model 4: Country S-Index
Good x Improved		3.02***(2.25-4.04)	2.95***(2.2-3.96)	2.95***(2.2-3.96)
Good x Worsened		1.75***(1.37-2.25)	1.73***(1.35-2.22)	1.73***(1.35-2.22)
Good x Same		1.61***(1.38-1.88)	1.61***(1.38-1.88)	1.62***(1.38-1.89)
Feeling anxious		0.95(0.84-1.07)	0.94(0.83-1.05)	0.94(0.83-1.06)
Feeling sad or depressed		1.14*(1.01-1.3)	1.13(1-1.29)	1.14(1-1.29)
Received home care before the pandemic		0.84(0.63-1.12)	0.85(0.64-1.13)	0.85(0.64-1.13)
Country-level covariates				
Level of volunteering before the pandemic		1.04***(1.02-1.06)	1.04***(1.02-1.06)	1.04***(1.02-1.06)
Standardised total COVID-19 cases per million		1.02(0.82-1.27)	0.99(0.8-1.22)	0.99(0.8-1.22)
Random intercept (country)	2.21***(1.4-3.47)	1.25**(1.08-1.45)	1.25**(1.08-1.44)	1.23**(1.08-1.41)
ICC	19.4%	6.4%	6.3%	5.9%

Table A3. Adjusted odds ratio (95% Confidence Interval) from multilevel logistic regression analysis of instrumental support provision (N= 36,518)

	Model 1: null model	Model 2: + control variables	Model 3: + COVID-19 exposure	Model 4: Country S-Index
Intercept	0.18***(0.15-0.22)	0.08***(0.05-0.11)	0.07***(0.05-0.11)	0.07***(0.05-0.1)
Covid-19 exposure on close ones			1.28***(1.19-1.39)	1.28***(1.19-1.39)
Covid-19 exposure on respondents			0.94(0.78-1.13)	0.94(0.78-1.13)
Country's standardised S-Index				1.13*(1.02-1.26)
Individual-level covariates				
Woman		1.07(1-1.13)	1.06(1-1.13)	1.06(1-1.13)
Age group				
50-59				
60-69		0.71***(0.64-0.77)	0.71***(0.64-0.77)	0.71***(0.64-0.77)
70-79		0.35***(0.31-0.39)	0.35***(0.31-0.39)	0.35***(0.31-0.39)
80+		0.18***(0.15-0.21)	0.18***(0.16-0.21)	0.18***(0.16-0.21)
Educational level				
Low				
Middle		1.31***(1.2-1.42)	1.3***(1.2-1.41)	1.31***(1.21-1.42)
High		1.42***(1.3-1.55)	1.4***(1.28-1.53)	1.41***(1.29-1.54)

	Model 1: null model	Model 2: + control variables	Model 3: + COVID-19 exposure	Model 4: Country S-Index
Good household SES		1.12**(1.03-1.21)	1.11*(1.02-1.2)	1.11*(1.02-1.21)
Changes in employment status				
Unemployed				
Became unemployed		1.43***(1.26-1.63)	1.42***(1.24-1.62)	1.42***(1.24-1.62)
Employed		1.21***(1.11-1.32)	1.2***(1.1-1.3)	1.20***(1.10-1.30)
Had instrumental support provision history		1.78***(1.67-1.9)	1.77***(1.66-1.89)	1.77***(1.66-1.89)
Living alone		1.17***(1.09-1.27)	1.18***(1.1-1.27)	1.18***(1.10-1.28)
Had frequent direct/online contacts		1.76***(1.64-1.88)	1.76***(1.65-1.88)	1.76***(1.65-1.88)
Had chronic condition before x during the pandemic				
No x No				
No x Yes		0.74*(0.59-0.95)	0.75*(0.59-0.95)	0.75*(0.59-0.95)
Yes x No		0.88***(0.82-0.94)	0.88***(0.82-0.94)	0.88***(0.82-0.95)
Yes x Yes		0.77**(0.64-0.93)	0.77**(0.64-0.94)	0.77**(0.64-0.94)
Self-rated health before x during the pandemic				
Poor x Improved		1.11(0.86-1.42)	1.1(0.86-1.41)	1.1(0.86-1.42)
Poor x Worsened		0.91(0.75-1.1)	0.91(0.75-1.1)	0.91(0.75-1.1)
Poor x Same				
Good x Improved		1.7***(1.35-2.14)	1.67***(1.33-2.11)	1.67***(1.33-2.11)
Good x Worsened		1.47***(1.24-1.74)	1.46***(1.23-1.73)	1.46***(1.23-1.73)
Good x Same		1.38***(1.26-1.51)	1.38***(1.26-1.51)	1.38***(1.26-1.51)
Feeling anxious		1.16***(1.07-1.25)	1.15***(1.07-1.24)	1.15***(1.06-1.24)
Feeling sad or depressed		1.05(0.97-1.14)	1.05(0.96-1.14)	1.05(0.96-1.14)
Received home care before the pandemic		0.74**(0.61-0.91)	0.75**(0.61-0.92)	0.75**(0.61-0.91)
Country-level covariates				
Level of instrumental support provision before the pandemic		1.01*(1-1.02)	1.01*(1-1.02)	1.01**(1-1.02)
Standardised total COVID-19 cases per million		1.26***(1.12-1.42)	1.23***(1.09-1.39)	1.24***(1.11-1.38)
Random intercept (country)	1.2**(1.06-1.35)	1.06**(1.02-1.1)	1.05**(1.01-1.1)	1.04*(1.01-1.07)
ICC	5.1%	1.6%	1.6%	1.2%

Table A4. Adjusted odds ratio (95% Confidence Interval) from multilevel logistic regression analysis of instrumental support receipt (N= 36,526)

Receiving instrumental support	Model 1: null model	Model 2: + control variables	Model 3: + COVID-19 exposure	Model 4: Country S-Index
Intercept	0.4***(0.32-0.5)	0.04***(0.02-0.08)	0.04***(0.02-0.08)	0.06***(0.03-0.13)
Covid-19 exposure on close ones			1.25***(1.15-1.35)	1.25***(1.15-1.35)
Covid-19 exposure on respondents			1.64***(1.38-1.95)	1.64***(1.38-1.95)
Country's standardised S-Index				0.69**(0.54-0.88)
Individual-level covariates				
Woman		1.55***(1.47-1.64)	1.55***(1.46-1.64)	1.55***(1.46-1.64)
Age group				
50-59				
60-69		2.13***(1.82-2.48)	2.13***(1.83-2.49)	2.13***(1.83-2.48)
70-79		4.66***(3.99-5.45)	4.74***(4.05-5.54)	4.73***(4.05-5.53)
80+		8.57***(7.28-10.08)	8.79***(7.47-10.34)	8.78***(7.46-10.33)
Educational level				
Low				
Middle		0.86***(0.81-0.92)	0.86***(0.8-0.92)	0.86***(0.8-0.92)
High		0.79***(0.73-0.85)	0.77***(0.72-0.84)	0.77***(0.72-0.84)
Good household SES		1.03(0.96-1.1)	1.02(0.95-1.1)	1.02(0.95-1.09)
Changes in employment status				
Unemployed				
Became unemployed		0.59***(0.49-0.71)	0.57***(0.48-0.69)	0.57***(0.48-0.69)
Employed		0.49***(0.43-0.54)	0.48***(0.42-0.53)	0.47***(0.42-0.53)
Had instrumental support receipt history		1.32***(1.23-1.4)	1.32***(1.23-1.4)	1.32***(1.23-1.4)
Living alone		1.51***(1.42-1.6)	1.52***(1.43-1.61)	1.52***(1.43-1.61)
Had frequent direct/online contacts		1.49***(1.41-1.58)	1.5***(1.42-1.58)	1.5***(1.42-1.58)
Had chronic condition before x during the pandemic				
No x No				
No x Yes		1.27*(1.06-1.52)	1.27*(1.06-1.53)	1.27*(1.06-1.53)
Yes x No		1.15***(1.08-1.22)	1.15***(1.08-1.21)	1.15***(1.08-1.21)
Yes x Yes		1.52***(1.34-1.73)	1.51***(1.33-1.72)	1.51***(1.33-1.72)
Self-rated health before x during the pandemic				
Poor x Improved		1.16(0.96-1.41)	1.13(0.93-1.37)	1.13(0.93-1.37)
Poor x Worsened		1.1(0.98-1.23)	1.09(0.97-1.22)	1.09(0.97-1.22)

**Receiving instrumental support**	**Model 1: null model**	**Model 2: + control variables**	**Model 3: + COVID-19 exposure**	**Model 4: Country S-Index**
Poor x Same				
Good x Improved		0.73**(0.57-0.92)	0.71**(0.56-0.91)	0.71**(0.56-0.91)
Good x Worsened		1.04(0.91-1.2)	1.01(0.87-1.16)	1.01(0.88-1.16)
Good x Same		0.69***(0.64-0.73)	0.69***(0.65-0.74)	0.69***(0.65-0.74)
Feeling anxious		1.33***(1.25-1.42)	1.32***(1.24-1.41)	1.32***(1.24-1.41)
Feeling sad or depressed		1.27***(1.18-1.35)	1.26***(1.18-1.35)	1.26***(1.18-1.35)
Received home care before the pandemic		3.44***(3.06-3.87)	3.46***(3.08-3.89)	3.46***(3.08-3.9)
Country-level covariates				
Level of instrumental support receipt before the pandemic		1.02(0.99-1.06)	1.02(0.99-1.06)	0.99(0.96-1.03)
Standardised total COVID-19 cases per million		1.32(1-1.76)	1.29(0.97-1.72)	1.14(0.89-1.45)
Random intercept (country)	1.24**(1.07-1.43)	1.3**(1.09-1.55)	1.3**(1.09-1.56)	1.19**(1.06-1.34)
ICC	6.0%	7.4%	7.5%	5.0%
